# Multimodality Images of a Mixed Atrial Septal Defect

**DOI:** 10.5935/abc.20160016

**Published:** 2016-02

**Authors:** Zafer Işılak, Uğur Küçük, Omer Uz, Murat Yalçın, Veysel Temizkan

**Affiliations:** 1 Department of Cardiology - Gulhane Military Medical Academy - Haydarpasa Training Hospital, Istanbul - Turkey; 2 Department of Cardiovascular Surgery - Gulhane Military Medical Academy - Haydarpasa Training Hospital, Istanbul - Turkey

**Keywords:** Atrial Septum / physiopathology, Heart Septal Defects, Atrial / physiopathology, Echocardiography, Three-Dimensional

**Figure 1:**
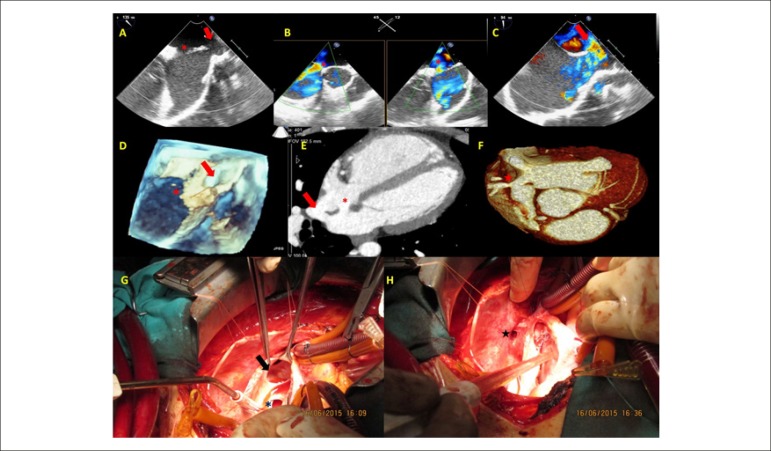
Multimodality and intraoperative images of a mixed atrial septal defect.

A 20-year-old male patient with dyspnea complaint was referred to our hospital. On physical examination there was grade 2/6 systolic murmur, which is best heard at the second left intercostal space and fixed split S_2_. ECG revealed sinus rhythm with complete right bundle brunch block. Transthoracic echocardiography demonstrated a secundum atrial septal defect (ASD) and Qp/Qs ratio was 1.6. The right ventricle was severely dilated, inconsistent with the size of the defect. Two-dimensional and color Doppler transesophageal echocardiographic examination confirmed the secundum type ASD (asterisk) and revealed an additional sinus venosus type ASD between the right atrium and the superior vena cava (SVC)(Figures 1A, 1B, 1C; Video 1). Three-dimensional transesophageal echocardiography verified both septal defects (Figure 1D). The patient underwent cardiac tomography for further anatomical outlining the. Figures 1E and 1F clearly show the secundum ASD (asterisk), sinus venosus ASD (arrow) and anomalous drainage of the right superior pulmonary vein to the SVC (star). The patient underwent surgery. Figures 1G and 1H show intraoperative images of the defects.

**Video:** Access the video through the link: http://www.arquivosonline.com.br/2016/english/10602/pdf/i10602013.pdf

The interatrial septum is anatomically divided into 5 septal zones. A mixed atrial septal defect involves 2 or more of the 5 septal zones and accounts for 7 of all atrial septal defects.^1^ In patients with severely dilated right ventricle and high Qp/Qs ratio, inconsistent with defect size, physicians should consider the presence of additional septal defects. These patients must be evaluated with advanced imaging modalities.
